# Tetracycline Susceptibility in *Chlamydia suis* Pig Isolates

**DOI:** 10.1371/journal.pone.0149914

**Published:** 2016-02-25

**Authors:** Manuela Donati, Andrea Balboni, Karine Laroucau, Rachid Aaziz, Fabien Vorimore, Nicole Borel, Federico Morandi, Edoardo Vecchio Nepita, Antonietta Di Francesco

**Affiliations:** 1 DIMES, Microbiology, University of Bologna, 40138, Bologna, Italy; 2 Department of Veterinary Medical Sciences, University of Bologna, Ozzano dell’Emilia, Bologna, Italy; 3 University Paris-Est, Anses, Animal Health Laboratory, Bacterial Zoonoses Unit, Maisons-Alfort, France; 4 Institute of Veterinary Pathology, Vetsuisse Faculty, University of Zurich, CH-8057, Zurich, Switzerland; 5 Parco Nazionale dei Monti Sibillini, Visso, Macerata, Italy; University of the Pacific, UNITED STATES

## Abstract

The aims of the present study were to assess the prevalence of *Chlamydia suis* in an Italian pig herd, determine the tetracycline susceptibility of *C*. *suis* isolates, and evaluate *tet*(C) and *tetR*(C) gene expression. Conjunctival swabs from 20 pigs were tested for *C*. *suis* by real-time polymerase chain reaction, and 55% (11) were positive. *C*. *suis* was then isolated from 11 conjunctival swabs resampled from the same herd. All positive samples and isolates were positive for the *tet*(C) resistance gene. The *in vitro* susceptibility to tetracycline of the *C*. *suis* isolates showed MIC values ranging from 0.5 to 4 μg/mL. *Tet*(C) and *tetR*(C) transcripts were found in all the isolates, cultured both in the absence and presence of tetracycline. This contrasts with other Gram-negative bacteria in which both genes are repressed in the absence of the drug. Further investigation into *tet* gene regulation in *C*. *suis* is needed.

## Introduction

*Chlamydiaceae* are obligate intracellular Gram-negative bacteria that cause a broad range of diseases both in humans and animals. *Chlamydia suis* infects pigs and has been associated with conjunctivitis, rhinitis, pneumonia, enteritis and reproductive disorders. Subclinical chlamydial infections are highly prevalent among pigs and make them more susceptible to other infections [1]. Both animal and human chlamydial infections are primarily treated with tetracycline or its derivatives. A stable tetracycline resistance phenotype was described for the first time in *C*. *suis* isolates from diseased and apparently healthy pigs in the US [2]. The resistance pattern was subsequently associated with *tet*(C) genomic islands integrated into the chlamydial chromosome and probably acquired through horizontal gene transfer [3]. Each island contained genes encoding a tetracycline efflux pump and a regulatory repressor (*tet*[C] and *tetR*[C], respectively), a *C*. *suis*-specific insertion element (IScs*605*) and additional genes involved in plasmid replication and mobilization [4]. Neither *tet*(C) nor *tetR*(C) was detected in the tetracycline sensitive reference S45 *C*. *suis* strain [3]. Studies performed on *C*. *suis* isolates from intensively reared Italian pigs showed a tetracycline resistance phenotype associated with the genomic island carrying the *tet*(C) resistance gene in several *C*. *suis* isolates [5]. *tet*(C)-positive *C*. *suis* strains have been reported in Swiss pig herds [6], and in Belgian, Cypriot and Israeli pig farms, where *in vitro* testing to tetracycline was also performed [7].

The aims of the present study were to assess the *C*. *suis* prevalence in an Italian pig herd, test *C*. *suis* isolates for their susceptibility to tetracycline, and correlate findings to the expression of the *tet*(C) and *tetR*(C) genes.

## Materials and Methods

### Sample collection and bacterial isolation

In March 2014, conjunctival swabs were collected at from 20 asymptomatic pigs aged 2–6 months reared in a pig herd located in Central Italy (42°48´20´´N 13°7´21´´E). Clinical history in this herd included conjunctivitis in piglets that had resolved spontaneously. A tetracycline treatment was routinely applied in the herd after castration of boars. For this study, no specific permission was required as the investigation was undertaken on the request of the owner for diagnostic investigation. The pig herd and the accessed land are privately owned and the owner gave permission to conduct the study on this site. All efforts were made to minimize the discomfort of the animals during sampling. This field study did not involve endangered or protected species. The sampling was performed by a veterinarian, gently rubbing a disposable cotton swab on conjunctival mucosa of both eyes. Approval by an animal ethics committee was not required as that the sampling did not involve blood.

In May 2014, conjunctival swabs were collected from 23 pigs reared in the same herd but from a litter different from the first sampling, to attempt chlamydial isolation according to Donati et al. [8]. Conjunctival swabs were placed in sucrose phosphate transport medium, transported at 4°C and processed immediately. Specimens were vortexed, then inoculated in duplicate onto LLC-MK2 cells (a continuous cell line derived from Rhesus monkey kidney tissue, provided by IZSLER Brescia, Italy), seeded in individual vials containing a glass coverslip at the bottom. After centrifugation at 800×g for three hours, the infected cell monolayers were incubated at 37°C for 48 hours and then fixed in methanol before detection of intracellular chlamydial inclusions by immunofluorescence technique.

### Bacterial DNA extraction

Genomic DNAs were extracted from the conjunctival swabs and the chlamydial positive cell cultures using the QIAamp DNA mini kit (Qiagen, Hilde, Germany), following the supplier's recommendations.

### Genus and species identification

Genomic DNAs extracted from the conjunctival samples were screened by a *Chlamydiaceae*-specific real-time polymerase chain reaction (rt-PCR) targeting a region of the 23S rRNA gene conserved among all *Chlamydiaceae* [9]. Samples with Ct values < 40 were considered positive and reanalyzed by a rt-PCR assay targeting a *C*. *suis*-specific region 23S rRNA gene [10]. All DNAs extracted from the Chlamydia-positive cell cultures were tested with the *C*. *suis*-specific rt-PCR.

DNAs from *C*. *suis*-positive samples and isolates were used as a template for a 1050-bp *omp*A gene fragment amplification [11]. The *omp*A amplicons were purified using a QIAquick PCR purification kit (Qiagen, Hilden, Germany) and both DNA strands were sequenced (Bio-Fab Research, Rome, Italy). The sequences obtained were compared with the public sequences available in GenBank using the BLAST server from the National Center for Biotechnology Information (http://blast.ncbi.nlm.nih.gov/Blast.cgi.).

### MIC determination

The *in vitro* susceptibility to tetracycline of the *C*. *suis* isolates was tested according to Donati et al. [12]. Antimicrobial susceptibility testing was performed with LLC-MK2 cells grown in 24-well plates with Eagle's minimum essential medium. Each of the 24-well plates was inoculated with 5 × 10^3^ inclusion-forming units (IFU) per milliliter. After centrifugation at 1,700 × *g* for 1 h, the medium was removed and replaced with medium containing different concentrations of antimicrobial drug. After incubation at 35°C for 48 h, infected monolayers were washed with phosphate-buffered saline (PBS), fixed with methanol, and stained for inclusions with a fluorescein-conjugated monoclonal antibody specific for the chlamydial lipopolysaccharide genus-specific antigen (Meridian Diagnostics, Inc., Cincinnati, OH, USA). The minimum inhibitory concentration (MIC) of tetracycline was defined as the lowest concentration preventing the detection of more than 90 per cent of the chlamydial inclusions compared with the drug-free control. All tests were run in triplicate.

### Molecular analysis of *tet* genes

A PCR amplifying a 608 bp fragment including the *tetR*(C) region and a PCR amplifying a 457 bp fragment of the *tetR*(C)-*tet*(C) region were performed on the *C*. *suis*-positive samples and isolates. The first PCR used the new primer *tet*R-F (5’-TTGGGGCAACCATTTCTGGT-3’) and the primer CS38 (5’-CCAAGGGATGACGACGACTG-3’) [3]. The second PCR was performed using the new primer *tet*RC-F (5’-TGCGTCGAGCAACGCACGCT-3’) and the primer CS43 reverted (5’-CAAAGCGGTCGGACAGTGCT-3’) [3]. In the first PCR, cycling conditions were as follows: 5 min of denaturation at 95° C and 35 cycles each consisting of denaturation at 94° C for 1 min, annealing at 58° C for 1 min and extension at 72° C for 1 min. A final elongation step of 5 min at 72° C completed the reaction. In the second reaction, PCR conditions were as described above, except for a higher annealing temperature (63°C). All amplicons were purified and sequenced for both DNA strands, as described above.

### Reverse transcription-PCR

Transcriptional analysis of *tetR*(C) and *tet*(C) genes was performed for all the *C*. *suis* isolates using a reverse transcriptase PCR (RT-PCR) performed on RNA templates extracted from *C*. *suis*-infected monolayers cultured in the absence or presence of tetracycline. A Swiss field *C*. *suis* isolate, named NB-1 [13] was included, as a negative control as it is *tet*(C)-PCR-negative and sensitive *in vitro* to tetracycline. RNA extraction was performed using the RNeasy Mini Kit (Qiagen, Hilden, Germany) following the supplier's recommendations. AMV Reverse Transcriptase (Promega, Madison, WI, USA) was used to catalyze the polymerization of DNA from RNA samples. PCRs for *tetR*(C) and *tet*(C) genes [3], targeting fragments of 450 bp and 525 bp, respectively, were performed on the complementary DNA (cDNA) obtained by the reverse transcription step.

## Results and Discussion

In the first sampling, 11 out of the 20 (55%) sampled pigs were positive by the *Chlamydiaceae*-specific RT-PCR and all were identified as *C*. *suis*. The *omp*A-amplicons from samples showed 96% sequence similarity (GenBank accession number KU668376) to the corresponding sequence of Rogers 130 *C*. *suis* strain (ATCC VR1482, GenBank accession number AF269277). In the second sampling, *C*. *suis* was successfully isolated from 11 out of 23 conjunctival swabs. The *ompA* sequences of the isolates were identical to each other and to sequences previously obtained from the *C*. *suis*-positive conjunctival swabs.

*In vitro* testing of the isolates to tetracycline showed MIC values ranging from 0.5 to 4 μg/mL (Table 1). In particular, MIC values were 0.5 μg/mL for one *C*. *suis* isolate (Eu-21), 2 μg/mL for six isolates (Eu-10/15/17/18/22/23), and 4 μg/mL for four isolates (Eu-4/7/8/19).

**Table 1:** *In vitro* MICs of tetracycline against 11 *Chlamydia suis* isolates.

No. isolates	MIC (μg/mL)
**1**	**0.5**
**6**	**2**
**4**	**4**

**Fig 1:**
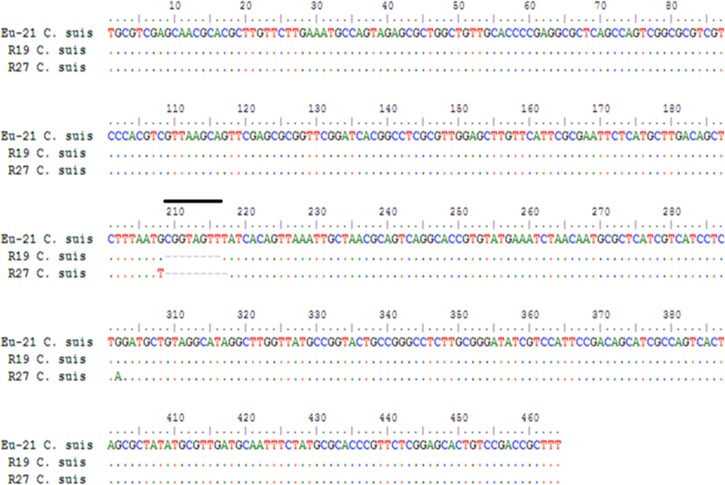
*TetR*(C)-*tet*(C) region of the Eu-21 *Chlamydia suis* isolate compared with the same genomic region of the R19 and R27 *C*. *suis* strains. The highlighted alignment shows the eight-nucleotide sequence deleted in R19 and R27 *C*. *suis* strains.

*TetR*(C) and *tetR*(C)-*tet*(C) amplicons were detected in all positive samples and isolates. *TetR*(C) and *tetR*(C)-*tet*(C) sequences (GenBank accession numbers KU668377 and KU668378, respectively) were identical to each other, and all they differed from those of the tetracycline-resistant R19 and R27 *C*. *suis* strains (GenBank accession number AY428550 and AY428551, respectively) described by Dugan *et al*. [3] as they presented a sequence of eight nucleotides in the *tetR*(C)-*tet*(C) intergenic spacer (Fig 1).

**Fig 2:**
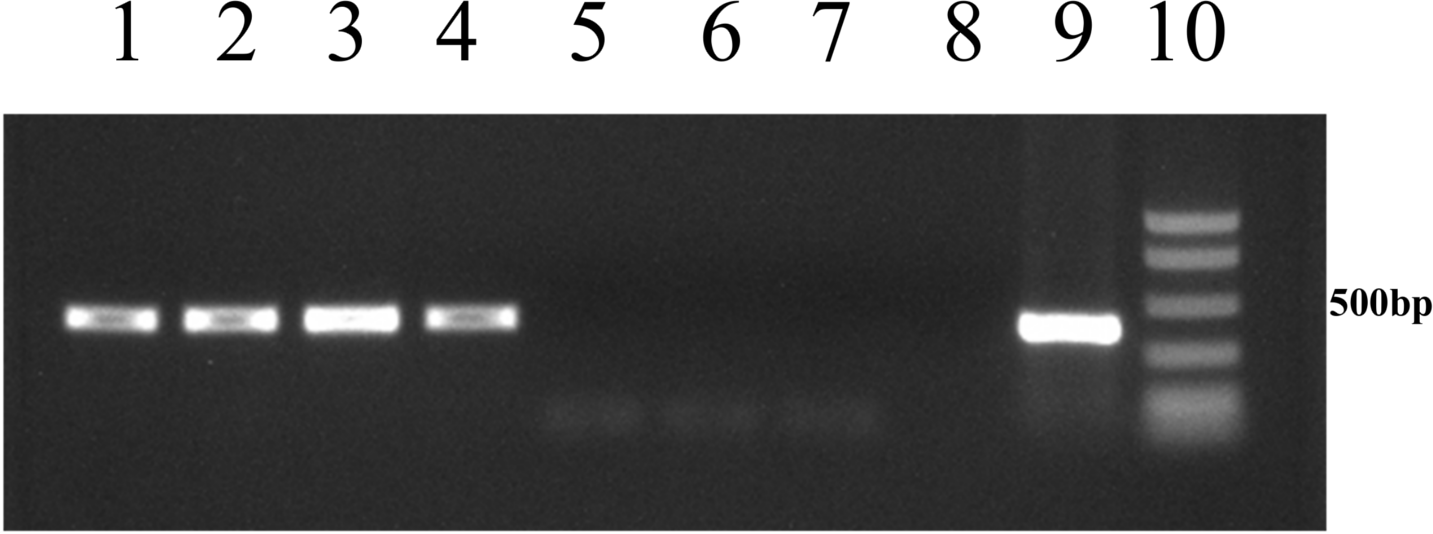
Analysis of *tetR*(C) transcription in Eu-22 and Eu-21 *Chlamydia suis* isolates. Lanes 1 and 2: *tetR*(C) transcript from Eu-22 in the absence and presence of tetracycline at a concentration of 0.25 μg/mL. Lanes 3 and 4: *tetR*(C) transcript from Eu-21 in the absence and presence of tetracycline at a concentration of 0.25 μg/mL. Lane 5: negative control using NB-1 *C*. *suis* isolate cultured in the absence of tetracycline. Lane 6: blank control of RNA extraction. Lane 7: blank control of reverse transcription. Lane 8: blank control of PCR. Lane 9: positive control using bacterial genomic DNA as a template. Lane 10: BenchTop Markers (Promega, Madison, WI, USA).

**Fig 3:**
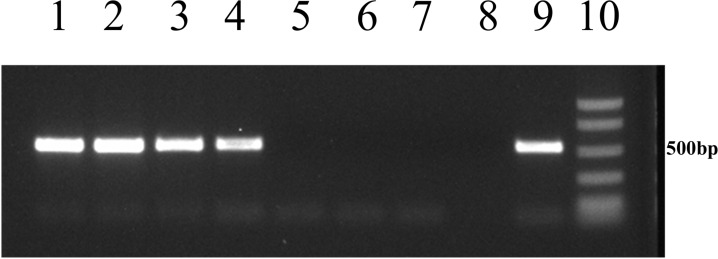
Analysis of *tet*(C) transcription in Eu-22 and Eu-21 *Chlamydia suis* isolates. Lanes 1 and 2: *tet*(C) transcript from Eu-22 in the absence and presence of tetracycline at a concentration of 0.25 μg/mL. Lanes 3 and 4: *tet*(C) transcript from Eu-21 in the absence and presence of tetracycline at a concentration of 0.25 μg/mL. Lane 5: negative control using NB-1 *C*. *suis* isolate cultured in the absence of tetracycline. Lane 6: blank control of RNA extraction. Lane 7: blank control of reverse transcription. Lane 8: blank control of PCR. Lane 9: positive control using bacterial genomic DNA as a template. Lane 10: BenchTop Markers (Promega, Madison, WI, USA).

*Tet*(C) and *TetR*(C) transcripts were detected in all the *C*. *suis* isolates cultured both in the absence and in the presence of tetracycline (Figs 2 and 3).

In recent years, the emergence of tetracycline-resistant *C*. *suis* strains has attracted increasing interest. Suchland *et al*. [14] demonstrated the *in vitro* horizontal transfer of tetracycline resistance from *C*. *suis* to clinical strains of *Chlamydia trachomatis*, an important human pathogen. In view of the potential public health concerns resulting from *in vivo tet*(C) resistance gene transfer from porcine chlamydial strains to human chlamydial pathogens, the spread of tetracycline-resistant *C*. *suis* strains needs to be monitored.

The endemic trend of *C*. *suis* infection in Italian pig herds found in the present study is comparable to previous reports [15], and the presence of *tet* genes proved widespread. Comparison of the *ompA* sequences from samples and isolates suggests the circulation of a single *C*. *suis* strain in the sampled herd, but the isolates showed a different degree of susceptibility to tetracycline, with MIC values varying from 0.5 to 4 μg/mL. For one isolate (Eu-21) the MIC was lower than the MIC values detected in the other isolatesand in previous reports on *C*. *suis* strains [3,7]. Interestingly, an MIC value of 0.5 μg/mL was detected in our previous study in two *tet*(C) PCR-positive *C*. *suis* isolates [5].

Previous studies showed that overexpression of the tetracycline resistance protein is lethal for *E*. *coli*, probably due to collapse of the membrane potential [16]. Therefore, the efflux-encoding determinants are strictly regulated. Tetracycline-inducible Tet repressor proteins turn down transcription of the resistance genes and of their own genes in the absence of the drug and allow expression of both proteins only in the presence of tetracycline [17]. Dugan *et al*. [3] emphasized that the *tet*(C) transcript was found in *C*. *suis* R19 cultured both in the absence and presence of tetracycline, whereas it was not detected in *E*. *coli* (pSC101) when cultured in a medium lacking tetracycline. The comparison between the nucleotide sequences of *C*. *suis* and *E*. *coli* showed that *tetR*(C) in R19 strain was truncated and that the operator region had an octanucleotide deletion relative to the homologous sequence in pSC101. The authors suggested that these differences may affect the regulation of *tet*(C) gene, eliminating the tight control placed on *tet*(C) expression in the absence of tetracycline.

The present study found *tet*(C) and *tetR*(C) transcripts in all *C*. *suis* isolates cultured in the absence and presence of tetracycline. All *C*. *suis* isolates showed the truncation of *tetR*(C), whereas the eight-nucleotide deletion upstream of the *tet*(C) start site was not present. These findings suggest that the presence or absence of the eight-nucleotide sequence does not affect regulation of the *tet*(C) gene in the absence of tetracycline. Our data confirm the unusual features of *tet* gene regulation and expression in *C*. *suis* compared to other Gram-negative bacteria. Further investigation on *tet* gene regulation in *C*. *suis* is needed.
